# Propensity score analysis of psychological intimate partner violence and preterm birth

**DOI:** 10.1038/s41598-022-06990-2

**Published:** 2022-02-21

**Authors:** Stella Martín-de-las-Heras, Khalid Saeed Khan, Casilda Velasco, Africa Caño, Juan de Dios Luna, Leticia Rubio

**Affiliations:** 1 Department of Forensic Medicine, School of Medicine, University of Málaga, Bulevar Louis Pasteur 32, 29017 Málaga, Spain; 2 Instituto de Investigación Biomédica de Málaga (IBIMA), Universidad de Málaga, Málaga, Spain; 3 Department of Preventive Medicine and Public Health, University of Granada, Granada, Spain; 4 Department of Midwifery, University of Jaen, Jaén, Spain; 5 Department of Obstetrics and Gynaecology, University of Granada, Granada, Spain; 6 Department of Biostatistics, University of Granada, Granada, Spain

**Keywords:** Paediatrics, Public health

## Abstract

Psychological intimate partner violence (IPV), a global public health problem, affects mothers during pregnancy. We evaluated its relationship with preterm birth. We established a cohort of 779 consecutive mothers receiving antenatal care and giving birth in 15 public hospitals in Spain. Trained midwives collected IPV data using the Index of Spouse Abuse validated in the Spanish language. Preterm was defined as birth before 37 completed weeks of gestation. Gestational age was estimated by early ultrasound. With multivariate logistic regression we estimated the relative association of IPV with preterm birth as adjusted odds ratios (AOR), with 95% confidence intervals (CI). In propensity score analysis, using weighting by inverse probability of exposure to IPV, the whole sample was used for estimating the absolute difference in probability of preterm amongst offspring born to mothers with and without IPV. Socio-demographic and other pregnancy characteristics served as covariates in both analyses. Preterm occurred in 57 (7.3%) pregnancies. Psychological IPV, experienced by 151 (21%) mothers, was associated with preterm birth (11.9% vs 6.5%; AOR = 2.4; 95% CI = 1.1–5.0; *p* = 0.01). The absolute preterm difference in psychological IPV compared to normal was 0.08 (95% CI = 0.01–0.16; *p* = 0.04). The probability of preterm birth was 8% higher on average in women with psychological IPV during pregnancy. As our analysis controlled for selection bias, our findings give credence to a causal inference. Screening and management for psychological IPV during pregnancy is an important step in antenatal care to prevent preterm birth.

## Introduction

Violence against women including intimate partner violence (IPV) is one of the most pervasive human rights abuses worldwide. IPV refers to behaviour within an intimate relationship that causes physical, sexual or psychological harm, including acts of physical aggression, sexual coercion, psychological abuse and controlling behaviours. This definition covers violence by both current and former spouses and partners^1,2^. Globally, the lifetime prevalence of IPV against women perpetrated by a male partner is around 30%^3^. In Europe, 32% of women reported experiencing psychological IPV and 43% reported psychological IPV including other forms of violence as controlling behavior, economic violence and blackmail with children^4^.

The consequences of IPV are magnified when the victim of violence is pregnant because of additional health risks to both the woman and her unborn child^1,5^. During pregnancy most women are in contact with the health care system making antenatal care a window of opportunity for identifying IPV. Violence during pregnancy is higher than many common obstetric conditions^6^ differing across countries and cultural settings^3,7–9^. In a European wide-survey, women who were pregnant during the relationship with their partner and who experienced violence in the relationship, 20% of the victims of current partner violence and 42% of victims of previous partner violence say that physical or sexual violence also took place during pregnancy^4^. A recent Spanish study documented that psychological IPV in pregnancy was reported by 21.0% of the women by using validated screening tools^10^. The awarness of this problem is high in Spain as political agreement to reduce gender violence (2017) was obtained with unanimity among all the political parties and supported by consistent provision of an annual budget. The Spanish agreement emphasizes that doctors, midwives and other allied healthcare professionals must act as active screeners, and that this should happen within a system where early detection is followed by proper multidisciplinary input^11^.

A worldwide estimation of 12.9 million children are born before 37 gestational weeks, which implies that 9.6% of all children being born preterm^12^ with devastating effects on the child's health^13^. IPV during pregnancy might contribute to preterm birth through an association with obstetric complications, e.g. preeclampsia, gestational diabetes^14–19^. Physical IPV during pregnancy has been most linked to preterm birth^20–24^. In a recent meta-analysis study, the psychological IPV was assessed in only 2 of the 30 articles^14^. However, psychological violence is also an important form of IPV but inadequate standardization of its measurement might difficult to quantify the health effects of this type of violence^16^. The deficiency of valid and reliable evaluations of IPV^16^ with frequent studies aiming exclusively on physical IPV^25^ have the result that the matter of psychological IPV during pregnancy mainly unnoticed.

It is increasingly being recognised that psychological victimization during pregnancy contributes to poorer overall health and temperament of the child. Psychological IPV during pregnancy have been linked to psychological outcomes such as high levels of stress, anxiety, and posttraumatic stress disorder^17,19^. Women who are victims of psychological IPV during pregnancy have been found to have more severe family/social problems and higher rates of psychiatric problems and comorbidity^9,15^. Consequently, psychological IPV may be a risk factor for negative pregnancy health experiences and behaviors including substance use and inadequate weight gain and prenatal care utilization, suggesting pathways by which psychological IPV may impact pregnancy and birth outcomes^15,17^. Continuous and improved investigation has been requested, especially because the harmful effects of non-physical IPV are underestimated^20,21,26,27^.

Observational studies^13,14,16,17,21^ of the link between psychological IPV and preterm birth are vulnerable to selection bias, a situation where certain characteristics related to the likelihood of exposure, e.g. socioeconomic status, can lead to an inaccurate estimate of the association, making a causal inference impossible. A thorough search of the literature showed that propensity score analysis has not been used to evaluate the relationship, reducing the risk of bias in the estimation of the association.

Considering the public health importance of IPV and preterm birth as important risk factors for maternal and infant morbidity, we examined if the experience of psychological IPV, perpetrated by current or former male partners, and captured with validated tools in pregnancy may be associated with preterm birth in a propensity score analysis to allow for evaluation of a causal inference.

## Methods

### Population, sample size and study subjects

A population-based study was designed for all public hospitals (n = 28) in Andalusia, Spain (number of births = 76,336). A cluster sampling approach was adopted to select 15 hospitals to represent service type (regional, n = 5; specialized, n = 10; and district, n = 13). A sample of 750 women, consecutively enrolling 50 women per hospital^8^, provided an accuracy of ± 2.5% with 99% confidence for IPV detection, assuming an IPV prevalence of 7.5%^16^ and an intraclass correlation coefficient of 5%^28^. Participants were women who received routine antenatal care giving birth in the hospital. Exclusion criteria were women with miscarriages, inability to converse in the Spanish language, and the presence of cognitive disease preventing collection. The study protocol was approved by the research ethics committees of all participating hospitals (Research Ethics Committees of Healthcare Hospitals, Healthcare Counselling, Andalusian Healthcare Service, Andalusian Government, Spain. Protocol code: VIO-EMB-AP-2017) in accordance with the “Ethical Principles for Medical Research Involving Human Subjects” adopted in the Declaration of Helsinki by the World Medical Association (64thWMA General Assembly, Fortaleza, Brazil, October 2013). All participants provided written informed consent prior to enrolment.

### Data collection procedures

Data were gathered during the immediate postpartum period by midwives at each hospital who were given specific training for participation in the study. Women were enrolled on successive days until the sample size per hospital was achieved (n = 50), preventing any day without sampling. Data were gathered in a room other than the ward in which the woman was hospitalized, where the study objective was explained, with guarantee that partner was not present. The strict anonymity and confidentiality of the information collected was guaranteed. Women participating signed informed consent. If the questionnaire responses evidenced IPV, the women were given comprehensive information on the police, judicial, and social resources available.

### Data collection instruments

#### Preterm birth

Preterm was defined as birth before 37 completed weeks of gestation. Gestational age was estimated by early ultrasound.

#### IPV Exposure

IPV was defined as physical, sexual, coercion or psychological violence, and controlling behaviours perpetrated by a current or past male partner^1,2^ during 12 months before giving birth. It was identified in the immediate postpartum period using Index of Spouse Abuse (ISA), a 30-item self-report instrument measuring the severity and frequency of abuse by weighted items (Supplementary Method)^29^. ISA (ranging from 0 to 100 points) measured two different types of violence: an ISA-P score that represents the severity of physical violence and an ISA-NP score that represents the severity of nonphysical or psychological violence. The higher scores reflect more severe IPV. Recommended cut-off scores were 10 for physical violence and 25 for psychological violence as at these thresholds the sum of false positives and false negatives was minimized^29^. The instrument was validated for application in Spanish^30^.

#### Socio-demographic measures

Data collected were age, nationality, schooling history, employment, relationship status, number of children, cohabitation with partner/family, and the availability of next of kin support (i.e. a relative who could be turned to when needed). Relationship status was coded as “married”, “committed relationship” but not married and “non-committed relationship” considered between individuals who may have casual sex without demanding or expecting the commitment of a formal relationship^10^.

#### Pregnancy intendedness

The women were asked: “At the time you became pregnant, did you want to become pregnant then, did you want to wait until later, did you want no (more) children, or did you not mind either way?” A pregnancy was considered unintended if the respondent stated that at the time, she became pregnant she would have liked to have waited until later to become pregnant (mistimed pregnancy) or that she did not want any (more) children (unwanted pregnancy)^31^.

#### Perinatal outcomes

Data was extracted from the prospectively documented individual health records during the pregnancy. Outcomes collected were: anaemia (< 10.5 g/dL), vaginal bleeding (threatened abortion and antepartum haemorrhage), stillbirth, urinary tract infection, vaginal infections (bacterial vaginosis, sexually transmitted infection, candidiasis, etc.), gestational diabetes (confirmed by glucose tolerance test at 24–28 weeks), gestational hypertension (> 140/90 mmHg), spontaneous preterm labour, low birth weight, others (e.g. hyperemesis, placental disorders, mental disorders, hypothyroidism and intrauterine growth retardation) and smoking in pregnancy.

### Statistical analysis

Multiple logistic regression analysis determined the relative association between IPV and preterm birth. The results were summarized as crude (COR) and adjusted odds ratios (AORs) with 95% confidence intervals (CIs). The absolute difference in probability of preterm amongst those with and without IPV was estimated using propensity score analysis^32^. Weighting by inverse probability of exposure to IPV was applied to the whole sample. The Average Treatment Effect (ATE) of psychological IPV vs no IPV on preterm birth was estimated for the entire sample. The covariates in both analyses (multiple regression analysis and propensity score analysis) were socio-demographic characteristics (age, relationship status, educational level, employment status, nationality, cohabitation, and kin support), desired pregnancy, number of children different from the actual birth, stillbirth, obstetric pathologies (any pathology during pregnancy except anemia or infections), infections during pregnancy and smoking in pregnancy. The covariates were selected based on previous results predicting IPV and on preterm birth theory^8^. The numbers of cases of physical IPV were too small for a reliable multivariate analysis.

## Results

**Figure 1:**
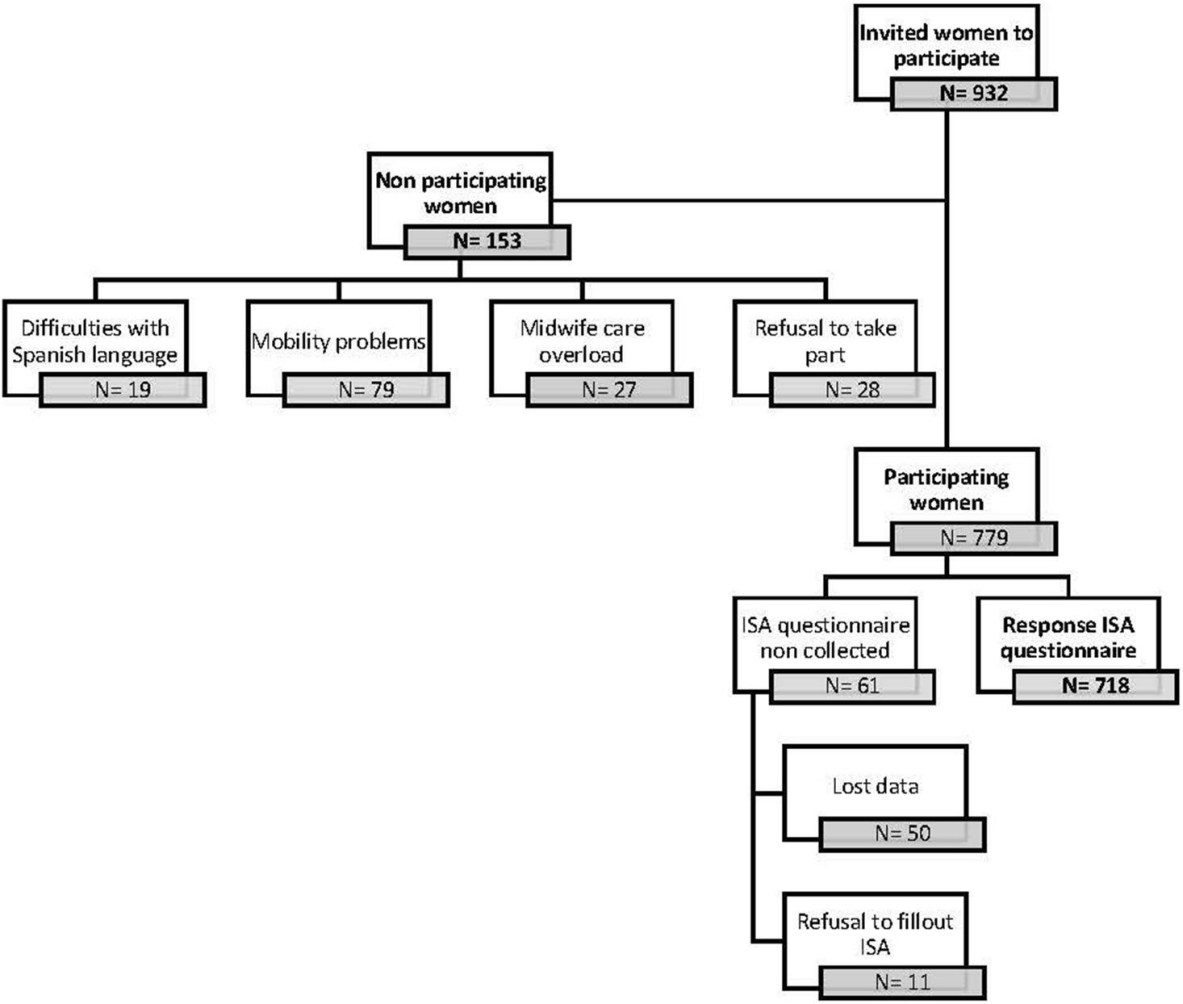
Flow diagram of the participants. Abbreviation: ISA, Index of Spouse Abuse.

The flow diagram of participants in this dataset shown in Fig. 1. The response rate amongst those invited to take part was 92.2% and the data loss was 4.3%: 28 women who refused to participate in the study and 11 who refused to fill out the ISA questionnaire (the latter were included in the study). IPV in pregnancy was reported by 21.3% (n = 153) of the women, including physical and psychological IPV, with no duplication of cases. Physical IPV was reported by 26 (3.6%) and psychological by 151 (21.0%).

The maternal socio-demographic characteristics and outcomes are shown in Tables 1 and 2. Preterm was observed in 57 (7.3%) of newborns; 11.9% among women who reported psychological IPV versus 6.5% among those who did not (COR = 1.9; 95% CI = 1.1–3.5; *p* = 0.02). The odds of preterm birth were higher in women with obstetric complications (COR = 4.3; 95% CI = 2.4–7.5) and those with a previous stillbirth (COR = 2.0; 95% CI = 1.1–4.5). In the adjusted analysis, the relative association of exposure to psychological IPV, compared to normal, with preterm birth remained significant (AOR = 2.4; 95% CI = 1.1–5.0; *p* = 0.01) (Table 2). The absolute difference in the probability of preterm birth among pregnant women who suffered psychological IPV vs those without exposure was 0.08 (95% CI = 0.01–0.16; *p* = 0.04), using propensity score analysis (Table 2).

**Table 1:** Socio-demographic characteristics of the sample.

	N	Fr (%)	Psychological IPV N(%)
**Age** (**yrs.)**			
< 20	26	3.7	12 (46.1)
20–24	95	13.6	29 (30.5)
25–29	187	26.8	43 (23.0)
30–34	260	37.2	39 (15.0)
35–39	104	14.9	18 (17.3)
≥ 40	26	3.7	5 (19.2)
**Relationship status**			
Married	466	65.1	67 (14.4)
Committed	102	14.2	27 (26.5)
Non-committed	148	20.7	56 (37.8)
**Years of schooling**			
< 7	262	36.5	68 (25.9)
7–12	350	48.8	72 (20.6)
> 12	105	14.6	11 (10.5)
**Employment status**			
Housewife	159	22.2	42 (26.4)
Unemployed	143	19.9	34 (23.8)
Employed	402	56.1	69 (17.2)
Student	13	1.8	5 (38.5)
**Nationality**			
Spanish	652	90.8	131 (20.1)
Other	66	9.2	20 (30.3)
**Cohabitation**			
Partner	657	91.5	126 (19.2)
Other	61	8.5	25 (41.0)
**Kin support**			
Yes	680	95.1	133 (19.6)
No	35	4.9	17 (48.6)

*IPV* Intimate partner violence.

**Table 2:** Univariate and multivariate regression models and propensity score analysis for preterm birth (< 37 weeks).

			Preterm birth	
	N (%)	R	OR (95% CI)	AOR (95% CI)
**Psychological IPV**		0.40*		
No (N = 567)	37 (6.5)		1	1
Yes (N = 151)	18 (11.9)		1.9 (1.1–3.5)*	2.4 (1.1–5.0)*
**Physical IPV**		0.03		
No (N = 692)	52 (7.5)		1	1
Yes (N = 26)	3 (11.5)		1.6 (0.5–5.5)	1.2 (0.2–6.1)
**Age (years)**		0.14		
< 20 (N = 31)	4 (12.9)		1	1
20–24 (N = 101)	12 (11.9)		0.9 (0.3–3.1)	0.7 (0.2–3.4)
25–29 (N = 200)	18 (9)		0.7 (0.2–2.1)	0.6 (0.1–2.9)
30–34 (N = 276)	13 (4.7)		0.3 (0.1–1.1)	0.2 (0.5–1.4)
35–39 (N = 120)	8 (6.7)		0.5 (0.1–1.7)	0.3 (0.5–2.0)
> 40 (N = 31)	2 (6.4)		0.5 (0.1–2.7)	0.4 (0.0–3.3)
**Schooling (years)**		0.03		
< 7 (N = 294)	24 (8.2)		1	1
7—12 (N = 377)	31 (8.2)		1.0 (0.6–1.8)	0.9 (0.5–1.7)
> 12 (N = 106)	2 (1.9)		0.2 (0.1–0.9)	EMPTY
**Employment**		0.12		
Housewife (N = 170)	11 (6.5)		1	1
Unemployed (N = 162)	19 (11.7)		1.9 (0.9–4.1)	2.0 (0.8 –5.0)
Employed (N = 430)	25 (5.8)		0.9 (0.4–1.9)	0.9 (0.4–2.3)
Student (N = 15)	2 (13.3)		2.2 (0.4–11.1)	0.5 (0.1–5.7)
**Nationality**		0.21		
Spanish (N = 710)	49 (6.9)		1	1
Other (N = 69)	8 (11.6)		0.6 (0.3–1.2)	0.8 (0.3–2.3)
**Relationship status**		0.02		
Married (N = 499)	36(7.2)		1	1
Committed (N = 106)	9 (8.4)		1.2 (0.6–2.6)	0.7 (0.3–1.8)
Non- committed (N = 171)	12 (7.0)		1.0 (0.5–1.9)	0.3 (0.1–0.8)*
**Cohabitation**		0.04		
Partner (N = 707)	50 (7.0)		1	1
Others (N = 71)	7 (9.9)		1.4 (0.6–3.3)	1.5 (0.4–5.1)
**Kin support**		0.01		
Yes (N = 738)	53 (7.1)		1	1
No (N = 37)	3 (8.1)		1.1 (0.3–3.8)	0.6 (0.2–2.6)
**Number of children**^**a**^		0.01		
0 (N = 399)	36 (9.0)		1	1
1 (N = 290)	17 (5.9)		0.6 (0.3–1.1)	0.5 (0.2–1.0)
≥ 2 (N = 90)	4 (4.4)		0.5 (0.2–1.4)	0.4 (0.1–1.4)
**Desired pregnancy**		0.04		
Yes (N = 656)	46 (7.0)		1	1
No (N = 118)	11 (9.3)		1.4 (0.7–2.7)	1.3 (0.5–3.2)
**Stillbirth**		0.11		
0 (N = 586)	36 (6.1)		1	1
1 (N = 151)	18 (11.9)		2.0 (1.1–3.8)*	2.2 (1.1–4.5)*
≥ 2 (N = 42)	3 (7.1)		1.2 (0.3–3.4)	1.3 (0.3–5.0)
**Obstetric pathologies**		0.26*		
No (N = 533)	21 (3.9)		1	1
Yes (N = 242)	36 (14.9)		4.3 (2.4–7.5)**	4.8 (2.4–9.2)**
**Infection during pregnancy**		0.05		
No (N = 440)	28 (6.4)		1	1
Yes (N = 331)	28 (8.5)		1.4 (0.8–2.3)	1.0 (0.5–1.8)
**Smoking in pregnancy**		0.01		
No (N = 639)	48 (7.5)		1	1
Yes (N = 137)	9 (6.6)		0.9 (0.4–1.8)	0.6 (0.3–1.5)

*IPV* Intimate partner violence, *OR* crude odds ratio, *AOR* adjusted odds ratio.

^a^Number of children different from the actual birth.

* p < 0.05.

** p < 0.01.

Propensity Score Analysis: ATE (Average Treatment Effect) = 0.08; 95% CI = 0.01–0.16; *p*= 0.04. Treatment effects (preterm birth): age, relationship status, schooling, employment, nationality, cohabitation, kin support, desired pregnancy, number of children, stillbirth.

## Discussion

In this study, psychological IPV, reported by 1 in 5 mothers, was associated with preterm birth. As the probability of preterm birth was 8% higher in women with psychological IPV, health care professionals should be alert about the risk to the offspring of women with psychological IPV exposure in pregnancy.

The strength of our investigation is based on the propensity score analysis that was used for the first time to draw causal inference between psychological IPV during pregnancy and preterm birth. The individual sampling weight contributes to the analysis using observed covariates in the whole sample^32^ to balance on average the measured socio-demographic and obstetric covariates among those with and without IPV. This reduced the risk of confounding in the relationship between the IPV exposure and preterm birth outcome. Even though this approach cannot control for unknown and unmeasured confounding it raises the possibility of a causal inference that metrics consideration. In future studies with larger samples, sensitivity analysis should be recommended to explore the bias due to unmeasured confounding variables. A key strength of our work is that it was a population-based study to identify psychological IPV during pregnancy with a validated tool (ISA) in the local language and midwives trained for data collection. Continuous ISA scores could be used in the logistic regression as an alternative approach in future research. The use of ultrasound scanning for gestational age determination was another strength of the study. This study presented data with a high response rate (> 90%). Despite the small figures, rejection to fill out the IPV instruments should always be considered methodological issues. Nevertheless, the low loss of ISA data (< 5%) should support the minimal influence on the validity of our results^8^. Further strength is that socio-demographic characteristics had no effect on preterm birth in the adjusted multivariate model. Perinatal outcomes**,** such as obstetric pathologies and a previous stillbirth were associated to preterm birth.

Although IPV has been also assessed in postpartum period^27^, that may be considered as one limitation of the study, since women tend to feel particularly vulnerable and may have induced an underreporting of the violence^33^. Another limitation is that the findings of this study may not generalize to non-Spanish populations, particularly to populations of pregnant women in countries with differing access to healthcare and/or quality of healthcare.

We found the prevalence of preterm 7.3% (< 37 weeks), the small numbers of extreme (< 28 weeks) and early (< 32 weeks) premature births did not allow us to draw any conclusion about preterm subgroups. Similar prevalence rates have been reported confirmed also by ultrasound scan^21^. However, maturity in a multi-country study, including 184 countries, showed that 11.1% of all deliveries were preterm^13^. The reported differences may reflect that pregnant women are exposed to different living conditions and also how accurately gestational age is determined^34^.

Women experiencing IPV during pregnancy were at increased risk for preterm birth ^14,16,17,21,22,27^ that is well established leading causes of neonatal morbidity and mortality^13^. However, it is important to note that several studies have not found a significant relationship between IPV and delivering preterm^23,35,36^. The lack of associations between IPV during pregnancy and preterm birth may be attributable to the small size of the sample studies. Similarly, generalizations are difficult to state among studies finding positive associations owing to different populations sampled, assessments, methods, and data analysis^17^.

The association between psychological IPV during pregnancy and preterm birth that we documented was adjusted for other known obstetric pathologies or a previous stillbirth. The causal inference, in addition to our inverse probability weighting analysis, is strengthened by several biological mechanisms. Psychological IPV during pregnancy may increase the risk of preterm birth through psychosocial or physical stress, depression, anxiety, isolation, decreased social support, and low self-esteem^14,16,17,37^. Indeed, psychological stress may induce pregnancy complications including such as preeclampsia or preterm labor or may aggravate preexisting conditions such as hypertension and gestational diabetes^17,37^. Psychosocial stress may also reflect in unhealthy behaviours during pregnancy such as smoking, or alcohol and drug consume, or may affect the adequate use of antenatal care services^14,16,17,37,38^. Regarding to the unhealthy behaviours, we found that smoking during pregnancy was not significantly associated to preterm birth. Besides, the stress of experiencing IPV during pregnancy may increase hypothalamic–pituitary–adrenal (HPA) activity. Higher levels of HPA hormones, including corticotrophin-releasing hormone (CRH), could initiate labor as well as restrict utero-placental perfusion^16,17^. Endothelial dysfunction and inflammatory cytokines all seem to be implicated in the pathogenesis of placental insufficiency, abruptio placentae and preterm birth^16^. Additional studies are needed to disentangle the independent and joint effect of IPV exposure and these risk factors on preterm risk.

Based on our findings, experiencing psychological IPV during pregnancy has a significant positive effect on premature birth. Thus, screening for non-physical IPV during pregnancy is an important step. There is a need for strengthening health care provision by involving other stakeholders who can support victims of psychological IPV by securing social and psychological support. Antenatal care represents an important opportunity to engage in preterm birth prevention through psychological IPV identification and management.
